# The School Nurses' Society

**Published:** 1902-07-19

**Authors:** 


// The Hospital

A Journal of

The Medical Sciences and Hospital Administration.

Vol. XXXII.—No. 825. Edited by Sir Henry Burdett, K.C.B., and Solomon C. Smith, M.D., M.R.C.P. [July 19, 1902.

### The School Nurses' Society.

In charity, as in other forms of well-doing, it is by no means unusual that the less conspicuous examples should, nevertheless, deserve to be numbered among the most useful; and we are disposed to think that a good illustration of this general truth is furnished by the modest activities of an almost unknown "society," on whose behalf a drawing-room meeting was held on Wednesday last at the house of Lady Windsor in Mount Street, kindly lent by her ladyship for the purpose. The "School Nurses' Society," the title of the charity in question, originated within the London School Board, and was established by the energy of Miss Honnor Morten, during her membership of that body. It is controlled by a small committee, presided over by the Hon. Lyulph Stanley, and composed chiefly of members of the Board, but with a few persons from outside; and it has for its object to provide for the regular visitation by nurses of elementary schools in the poorest districts of the metropolis, for the purpose of giving necessary attention to a great variety of troubles from which the children resorting to them are liable to suffer—troubles often apparently insignificant in any individual case, but none the less amounting collectively to a very formidable and dangerous total.

In the families of the better paid and more intelligent wage earners of the metropolis it may be said, as a general truth, that the children are very kindly treated and very carefully looked after, in so much that few sights are more pleasing than those furnished by the family groups which may be seen on Sundays and holidays, in all open spaces or places of public resort. So much is this the case that, a few years ago, when a deputation of German workmen came to this country, deputed by one of their trade societies to report upon the conditions of English labour, their own more rudimentary civilisation was much offended by what they saw, and they described in strong lauguage the unmanliness (*die Unmännlichkeit*) of the English workman, who, after a day's outing, might be seen carrying tired children home, instead of leaving that task to the mothers. If their researches had extended to the class of unskilled workers, earning but a precarious subsistence, they would have found the prevalence of methods bearing more resemblance to their own; for in the families of such people children are too often apt to be treated with harshness and neglect.

For the harshness, in such cases, there can probably be no other remedy than such as will in time be furnished by the gradual growth of a more healthy state of public opinion among the classes chiefly concerned; but the aim of the School Nurses' Society is in some measure to obviate the consequences of neglect. In the poorest districts of London, children come to the elementary schools suffering from all sorts of minor maladies and injuries, and sometimes from the beginnings of serious disease. They are often verminous, they have broken chilblains, their badly shod feet have been hurt by stones or wounded by broken glass, they have cut and often festering fingers, or bruises, or tarsal ophthalmia, and nothing is done for them at home. Even when not serious, the pain of such conditions places an additional difficulty in the way of giving the attention necessary for learning; while it often happens that a sore throat may in reality be diphtheria, or that a supposed cold may be measles, conditions in which prompt action is required for the safety of other children, as well as for that of the first invalids. Abundant evidence is now forthcoming to show that elementary schools are the chief causes of the diffusion of infectious diseases; and it is obvious that the teachers, trained only to attend to their proper work, and fully occupied in its performance, cannot always be on the alert with regard to cases of illness. Under the society to which we are referring, trained nurses are employed to visit certain schools at regular intervals, and it is found that the teachers and the children gladly cooperate in bringing under their notice every case in which their services seem to be required. The nurse is more likely, of course, than either an ignorant mother or a pre-occupied teacher to recognise the beginnings of serious illness and to insist upon the necessity of medical advice being procured. She cleanses and dresses the cuts and wounds which, small as they may be, are certainly painful, and are possible causes of serious or fatal erysipelatous inflammation, and she shows how they may best be protected against injury or septic invasion. It is manifest that the attention bestowed upon these children can have nothing but a good influence upon their characters. The worse are the domestic influences by which they are surrounded, the more important it is to awaken in their small minds a consciousness of being cared for by people more fortunate than themselves, and of having their places in the world in which they find themselves. The Society is much hampered by the smallness of its income; and it seeks but the modest sum of five hundred pounds a year in order to deal with the urgent demands upon its activity. It is difficult to conceive that the money could be better bestowed.

